# Autologous Hematopoietic Stem Cell Transplantation for Behçet’s Disease: A Retrospective Survey of Patients Treated in Europe, on Behalf of the Autoimmune Diseases Working Party of the European Society for Blood and Marrow Transplantation

**DOI:** 10.3389/fimmu.2021.638709

**Published:** 2021-05-06

**Authors:** Mathieu Puyade, Amit Patel, Yeong Jer Lim, Norbert Blank, Manuela Badoglio, Francesca Gualandi, David D. Ma, Natalia Maximova, Raffaella Greco, Tobias Alexander, John A. Snowden

**Affiliations:** ^1^ CHU de Poitiers, Service de Médecine Interne et Maladies Infectieuses, Poitiers, France; ^2^ CHU de Poitiers, CIC-1402 Poitiers, France; ^3^ Haematology and Transplant Unit, The Christie NHS Foundation Trust, Manchester, United Kingdom; ^4^ Haemato-Oncology Department, Clatterbridge Cancer Centre, University of Liverpool, Liverpool, United Kingdom; ^5^ Department of Hematology, Oncology and Rheumatology, Internal Medicine V, University Hospital of Heidelberg, Heidelberg, Germany; ^6^ EBMT ADWP Office, Paris, France; ^7^ U.O. Ematologia Centro Trapianti Midollo - Ospedale Policlinico San Martino, Genova, Italy; ^8^ Department of Haematology and BM Transplantation, St Vincent’s Hospital Sydney and St Vincent’s Clinical School, Faculty of Medicine, University of New South Wales (UNSW) Sydney, Sydney, NSW, Australia; ^9^ Institute for Maternal and Child Health – IRCCS Burlo Garofolo, Trieste, Italy; ^10^ Haematology and Bone Marrow Transplant Unit, IRCCS San Raffaele Scientific Institute, Milan, Italy; ^11^ Vita-Salute San Raffaele University, Milan, Italy; ^12^ Department of Rheumatology and Clinical Immunology, Corporate Member of Freie Universität Berlin, Humboldt-Universität zu Berlin, and Berlin Institute of Health, Charité - Universitätsmedizin Berlin, Berlin, Germany; ^13^ Deutsches Rheuma-Forschungszentrum (DRFZ), an Institute of the Leibniz Association, Berlin, Germany; ^14^ Sheffield Teaching Hospitals NHS Foundation Trust, Sheffield, United Kingdom

**Keywords:** Behçet’s disease, autologous stem cell transplantation, efficacy, toxicity, immune reset

## Abstract

**Background:**

Behçet’s Disease (BD) is an autoimmune disease mostly presenting with recurrent oral and genital aphthosis, and uveitis. Patients are rarely refractory to immunosuppressive treatments. Autologous hematopoietic stem cell transplantation (aHSCT) is a standard of care in other autoimmune diseases. Some patients with BD have been treated with aHSCT based on compassionate use.

**Objectives:**

Evaluate the outcome of aHSCT in adult patients with BD treated in member centers of the European Society for Blood and Marrow Transplantation (EBMT).

**Methods:**

Adults who received aHSCT primarily for BD were identified retrospectively in the EBMT registry and/or in published literature. Data were extracted from either medical records of the patient or from publications.

**Results:**

Eight out of 9 cases reported to the registry and extracted data of 2 further patients from literature were analyzed. Four were female, median age at onset of BD was 24y (range 9-50). Median age at aHSCT was 32y (27-51). Patients had received median 4 (2-11) previous lines of therapy (89% corticosteroids, 50% methotrexate, anti-TNFα therapy or cyclophosphamide). All patients had active disease before mobilization. Conditioning regimen was heterogeneous. Median follow-up was 48 months (range 6-240). No treatment-related mortality was reported. This procedure induced complete remission (CR) in 80%, partial remission in 10% and lack of response in 10% of the patients. Relapse rate was 30% (2 relapses in patients in CR and 1 relapse in the patient in PR) with panuveitis (n=1), aphthosis (n=2) and arthralgia (n=1). Six patients were in CR. No late complications were reported.

**Conclusion:**

aHSCT has an acceptable safety profile and represents a feasible and relatively effective procedure in severe and conventional treatment-resistant cases of BD and has the potential to stabilize BD in patients with life-threatening involvements.

## Key points

1. aHSCT has an acceptable safety profile and represents a feasible and relatively effective procedure in severe and conventional treatment-resistant cases of BD
2. aHSCT has the potential to stabilize BD in patients with life-threatening involvements.
3. The rate of failure and relapse seems to be low.

## Introduction

Behçet´s disease (BD) is a rare autoimmune disease mostly presenting with recurrent oral and genital aphthosis, and uveitis. Other common symptoms include gastrointestinal, vascular, neurological and articular manifestations (1). The first internationally agreed criteria for BD were the International Study Group (ISG) criteria (2). They had very high specificity, but lacked satisfactory sensitivity, missing out on a sizable subset of patients. The International Criteria for Behçet’s Disease (ICBD) were created in 2006 to overcome this lack of sensitivity. This classification was revised in 2006. A score ≥ 3 is associated with sensitivity of 95% and specificity of 90.5% (3). Treatment is based on chronic immunosuppression with conventional disease-modifying or targeted biologic drugs such as corticosteroids, intravenous cyclophosphamide, cyclosporine or anti TNFα therapy (4). The most recent European League Against Rheumatism (EULAR) recommendations were updated in 2018 (5). They did not mention autologous hematopoietic stem cell transplantation (aHSCT), possibly due to limited specific data in BD.

Over the past 20 years, aHSCT has become a standard of care for early rapidly progressive Systemic Sclerosis (6–11) and has emerged as a promising treatment modality for severe Multiple Sclerosis (12–14) and Crohn’s Disease (12, 15). But patients with rarer diseases, such as BD, have undergone aHSCT based on compassionate use.

Led by the Autoimmune Diseases Working Party (ADWP) of the European Society for Blood and Marrow Transplantation (EBMT), a previously published retrospective analysis of HSCT covered all vasculitis (16), in which partial and complete responses were reported for two patients with BD. Other publications on autologous stem cell transplantation for BD [without a concomitant hematologic malignancy, as it has also been reported (17, 18)] only consist of case reports (19–23). Only one failure with aHSCT in BD has been reported (22). Further data and composite analysis are required to inform the balance of benefits and risks of aHSCT in patients with severe, refractory BD. The aim of this retrospective study was therefore to bring together a larger experience in order to describe the efficacy and toxicity of aHSCT in adults with BD treated in EBMT centers.

## Methods

### Study Design

The European Society for Blood and Marrow Transplantation (EBMT) is a medical and scientific organization that represents HSCT centers in Europe and worldwide. The EBMT registry contains details on transplants performed for auto-immune diseases since 1986, including over 3000 patients undergoing HSCT for autoimmune diseases. Patients provided written consent before transplant for the collection and analysis of anonymized data, and data were maintained in the central EBMT registry in line with legal and regulatory requirements. This is a multicenter retrospective study approved by the Autoimmune Diseases Working Party (ADWP) of the EBMT. Once eligible patients had been identified from the database, data already held within EBMT records were sourced and referring centers were asked to complete a questionnaire to provide further detailed information regarding patients’ pre- and post-transplant disease status. All relevant data were collated and held in a specially designed database within the EBMT. Data from published cases treated in Europe were extracted from the literature in PubMed and Science Direct. The algorithm was (“Behcet Syndrome”[Mesh]) AND “Hematopoietic Stem Cell Transplantation”[Mesh] in Pubmed and “Behcet” AND “Stem Cell transplantation” in Science Direct.

### Patient Eligibility

Inclusion criteria were:

1. Behçet’s disease (BD) diagnosed according to International Criteria for Behçet’s disease (ICBD) classification criteria
2. Aged 18 years or older at the time of treatment with autologous stem cell transplantation treated in an EBMT member center

Exclusion criteria were:

1. aHSCT for a concomitant disease in patients with a diagnosis of Behcet’s disease
2. Patients with follow-up <6 months

### Definitions of Disease Activity

The following definitions of disease activity and response were used:

- Active disease (persisting or recurring disease activity post-transplantation, insufficiently controlled by glucocorticoids and/or disease-modifying drugs).
- Drug-dependent partial response (DDPR) (any documented clinical and/or laboratory response in patients receiving glucocorticoids at a dose equivalent or greater than 7.5mg of prednisolone/day and/or disease-modifying drugs).
- Drug-dependent complete response (DDCR) (no evidence for disease activity in patients receiving glucocorticoids at a dose equivalent or greater than 7.5mg of prednisolone/day and/or disease-modifying drugs)
- Complete remission (CR) (no evidence for disease activity in patients with glucocorticoids at a dose below 7.5mg of prednisolone and absence of disease-modifying drugs).

A major relapse was considered in case of involvement of a life-threatening (pulmonary or vascular event for example) or functional-threatening organ (which was mainly eye). Other instances were considered as a minor relapse.

### Study Endpoints

Primary endpoint was response to treatment at the last follow-up visit. Progression was defined as absence of improvement but also as relapse of the disease requiring reintroduction or re-escalation of immunosuppression.

Secondary endpoints were:

- overall survival,
- relapse rate, defined as recurrence or new development of ulcerative disease, ocular lesions or vascular event,
- number of patients in CR, DDPR, DDCR, Active disease at last follow-up visit,
- toxicities related to aHSCT,
- time to reintroduction or re-escalation of immunosuppressive treatment,
- Non-Relapse Mortality (NRM) at 100 days and 1 year, defined as any death following transplant that cannot be attributed to active disease.

### Statistical Method

Due to the low number of patients and the heterogeneity of the population that do not allow any adjustment, statistics were only descriptive and no comparison was performed. Categorical variables were described as numbers and percentage and continuous variables using median and range.

### Data Collection

Cases for inclusion in the study were identified by database queries in the EBMT Registry according to the abovementioned inclusion criteria. A letter of invitation to participate in the study was sent to the EBMT centers. Data were extracted locally retrospectively: demographic and general data (age at BD onset, sex, age at aHSCT, ICBD criteria, number of previous therapies before aHSCT l304 and l306, daily Prednisolone dosage at baseline, therapies prior to HSCT), mobilization regimen, transplant data (graft manipulation, stem cell yield and number of collections, conditioning regimen, Time to engraftment, Adverse event before 100 days), late complications (after day 100), BD status at mobilization, 6 months, 12 months, and yearly post-aHSCT, date of most recent follow-up, date of reintroduced immunosuppression with time-point of reintroduction, relapse or progression, survival status, cause of death if happened. In case of extraction of the case from the literature, the same data were collected.

## Results

### Selection

**Figure 1:**
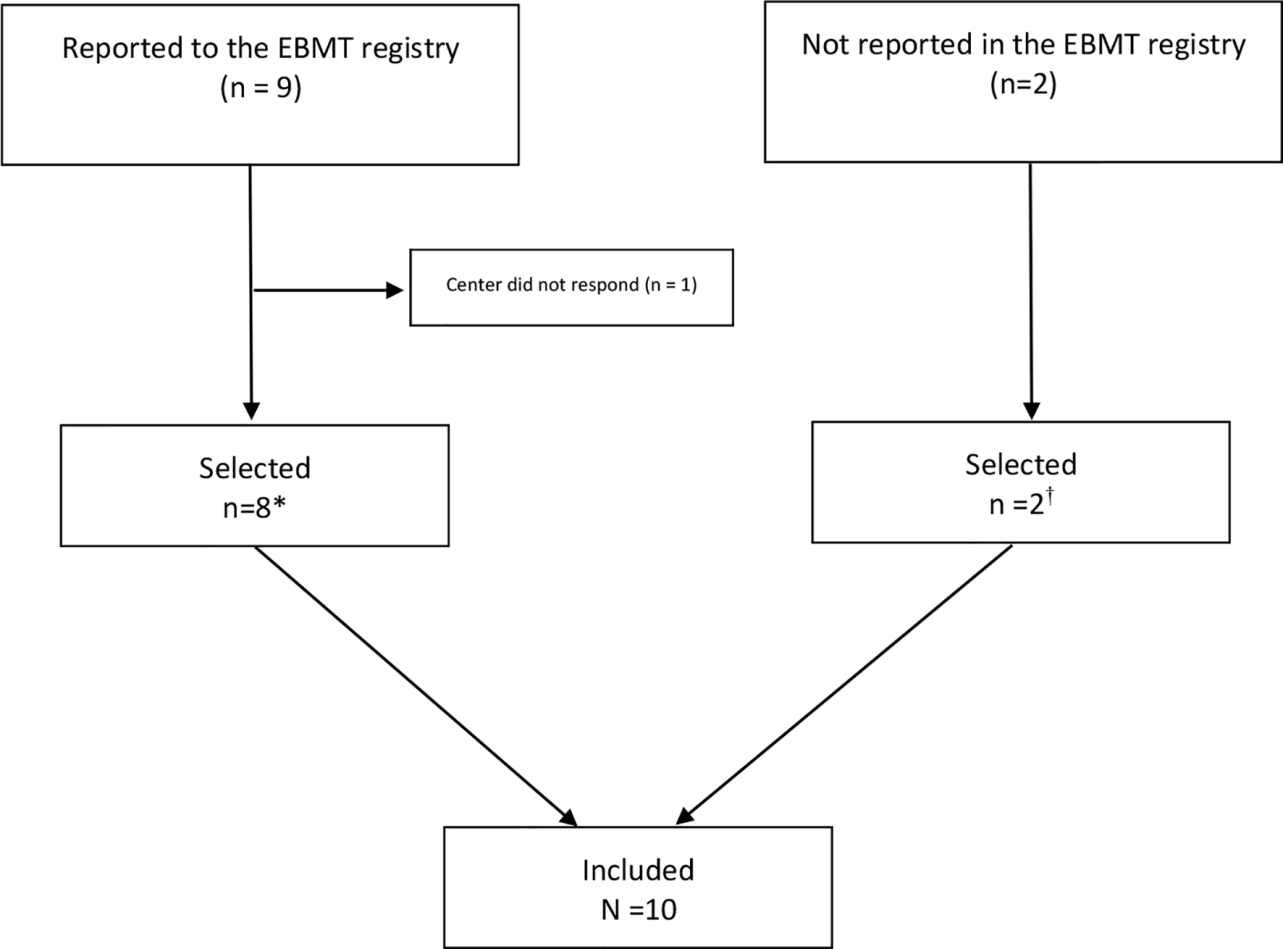
Flow Chart on identification of patients treated with autologous hematopoietic stem cell transplantation for Behçet’s Disease in European Society of Blood and Marrow Transplantation (EBMT) centers (N=10). *Preliminary results of two patients with shorter follow-up were previously published (23). Long-term follow-up data are included in this manuscript. ^†^Two cases were already published: De Cata A, Intiso D, Bernal M, et al: Prolonged Remission of Neuro-Behcet Disease following Autologous Transplantation. Int J Immunopathol Pharmacol 20:91–96, 2007 (center of Foggia).

Nine patients were registered in the EBMT database who all signed informed consent. Data were available for 8 patients [two cases were already published but with shorter follow-up (23)]. Two patients were treated in an EBMT center but not reported in the registry. They were identified from the published literature (20). All in all, 10 patients were included in our study (**Figure 1**).

### Patient Characteristics

Demographic and clinical characteristics of patients are provided in **Table 1**. All patients from EBMT registry had an ICBD score greater than 4 points. For the 2 patients extracted from the literature, diagnosis was made according to ISG criteria. Four patients were female. Median age at the onset of the disease was 24 years (range 9-50y), and median age at the transplantation was 32 years (range 26-51y). Patients had received median 4 (2-11) previous lines of treatment: 89 % of the patients were treated with corticosteroids, and 50 % received either methotrexate, anti-TNFα therapy or cyclophosphamide. All patients had active disease before aHSCT (one missing data). Main indications were severe uncontrolled and usually multiple involvements: ocular (4 patients) gastro-intestinal (4 patients), neurological (3 patients), cardiac (2 patients), lung and kidney (1 patient). Aphtosis was not controlled in 4 patients. For one patient, indication of transplantation is unknown. Detailed observations are presented in **Table 2**.

**Table 1:** Characteristics of the patients and outcomes.

	Median (range) or %	Missing data
	(N=10)	
**Characteristics of the patients**
** Sex (Female)**	40% (N=4)	0
** Age at onset (years)**	24 (9-50)	0
** Age at transplantation (years)**	32 (26-51)	0
** Number of previous lines of treatment**	4 (2-11)	0
** Active disease before mobilization**	100%	1
**Mobilization**		1
** Cyclophosphamide + G-CSF**	8	
** G-CSF alone**	1	
** Dose of cyclophosphamide during mobilization**	2 (1.5-4)	0
**Conditioning regimen**		0
** Melphalan 200mg/m²**	5	
** Cyclosphosphamide 200 mg/kg –ATG 4.5 mg/kg**	2	
**BEAM**	3	
** Number of days before engraftment showing:**		
** Neutrophils > 0.5 x10^9^/L**	11.5 (6-13)	0
** Platelets > 50 x10^9^/L**	14 (13-20)	1
**Complications before Day +100**		
** Fever of Unknown Origin**	2 patients/2 episodes	
** Infectious**	2 patients/2 episodes	
** Other:**	1 patient/3 episodes	
** Atrial Fibrillation, Line associated DVT, Depression**		
**Complications after Day 100**	0	0
**Overall Response**		0
** CR**	8	
** DDPR**	1	
** No response**	1	
** Major Relapse (n=1)**		0
** Uveitis**	1	
** Minor Relapse (n=2)**		0
** Aphtosis**	2	
** Arthralgia**	1	
**Median Follow-up (months)**	48 (6-252)	0

ATG, Anti Thymocyte Globulin; BEAM, BCNU – Etoposide-Aracytine- Melphalan; CR, Complete Remission: no evidence for disease activity in patients with daily prednisolone equivalent dosage ≤7.5mg and absence of disease-modifying drugs; DVT, Deep Venous Thrombosis; DDPR, Drug-Dependent Partial Response i.e. any documented clinical and/or laboratory response in patients receiving glucocorticoids ≥7.5mg daily prednisolone dosage and/or disease-modifying drugs. G-CSF, Granulocyte-colony stimulating factor.

Table 2Individual characteristics and outcome for patients with Behçet’s disease treated with autologous stem cell transplantation in European Society of Blood and Marrow Transplantation centers (N=10).

**Table:** 

a) Before transplantation							
Patient****	Center	Sex	Age at onset	Organ involvement	Previous treatments	Status of the disease before transplantation	Indication of transplantation
**1**	Trieste	F	29	G,O, Oc, V, GI	TS, SC, CSA, AZA	Active	G, O, Oc, V, GI
**2**	Heidelberg	M	48	G,O, Oc, V, N, K, L	SC, iCYC, oCYC	Active	G, O, Oc, K, L
**3**	Heidelberg	M	25	G,O, S, V, CI	SC, MTX	Active	G, O, Oc, CI
**4**	Foggia	M	22	N*	SC, CLB	Active	N
**5**	Foggia	M	23	N*	SC, CLB	Active	N
**6**	Genova	F	20	G,O, Oc, S, A, N, GI	TS, SC, iCYC,	Active	N, GI
**7**	Sydney	F	16	G,O, Oc, S	SC, MTX, IFN, IFX, RTX, PYX, oCYC, CSA, TCZ	Active	Oc
**8**	Liverpool	M	24	G,O, Oc, S, V	SC, MTX, IFX, iCYC, TCL, MMF, ETN	Unknown	Unknown
**9**	Liverpool	F	9	G,O, S, P , A, GI	TS, SC, COL, DAP, MTX, IFX, ADA, ATZ, AZA, LF, ETN	Active	O, GI
**10**	Liverpool	M	50	G,O, S, V, A, N, GI, CI	IFN, ATZ, AZA, MMF, ETN, CTZ	Active	V, GI, CI

*patients extracted from the literature, fulfilling International Study Group criteria of Behcet’s Disease. A, Arthritis; ADA, Adalimumab; ATZ, Alemtuzumab; AZA, Aziathoprine; CI, Cardiac Involvement; CLB, Chlorambucil; COL, Colchicine; CSA, Cyclosporine; CTZ, Certolizumab; DAP, Dapsone; ETN, Etanercept; FU, Follow-up; iCYC. intravenous Cyclophosphamide; IFX, Infliximab; IFN, Interferon alpha; G, Genital aphtosis; GI, Gastro-Intestinal Involvement; K, Kidney; L, Lung; LF, Leflunomide; MMF, Mycophenolate Mofetil; MTX, Methotrexate; N, Neurological involvement; O, Oral aphtosis; Oc, Ocular involvement; oCYC, Oral Cyclophosphamide; P, Pathergy test positive; PYX, Pentoxifylline; RTX, Rituximab; S, Skin involvement (skin ulcer or erythema nodosum); SC, Systemic Corticoids; TCL, Tacrolimus; TCZ, Tocilizumab; TS, Topical Steroids.

**Table 2B:** 

b) Characteristics of the transplantation and outcome.								
Patient	Year of transplant	Age at transplant	Mobilization	Conditioning regimen	Complications before D100	OR	FU in this study(months)	Case previously published (FU reported in months)
1	1999	33	G-CSF	iCYC+ATG		CR	48	No
2	1999	49	iCYC + G-CSF	Melphalan	Fever of unknown origin	CR	252	Yes (96)
3	1999	31	iCYC + G-CSF	Melphalan	Fever of unknown origin	CR	252	Yes (72)
4	Before 2002	26	iCYC + G-CSF	BEAM		CR	48	Yes (48)
5	Before 2002	26	iCYC + G-CSF	BEAM		CR	48	Yes (48)
6	2002	30	iCYC + G-CSF	BEAM		NR	6	No
7	2011	49	iCYC + G-CSF	iCYC+ATG		DDPR^*^	48	No
8	2009	29	iCYC + G-CSF	Melphalan		CR^*^	24	No
9	2016	42	iCYC + G-CSF	Melphalan	Pneumonia (Rhinovirus + Klebsiella Pneumoniae), AF, Line-DVT, Depression	CR^*^	37	No
10	2016	51	iCYC + G-CSF	Melphalan	Pneumonia	CR	96	No

*Relapse.

AF, Atrial Fibrillation; ATG, Anti Thymocyte Globulin; BEAM, Bicnu, Etoposide, Aracytine, Melphalan; CR, Complete Remission: no evidence for disease activity in patients with daily prednisolone equivalent dosage ≤ 7.5mg and absence of disease-modifying drugs; DDPR, Drug- Dependent Partial Response = any documented clinical and/or laboratory response in patients receiving glucocorticoids ≥7.5mg daily prednisolone dosage and/or disease modifying drugs; DVT, Deep Venous Thrombosis; iCYC, intravenous Cyclophosphamide; G-CSF, Granular Colony Stimulating Factor; NR, No Response; OR, Overall Response.

### aHSCT

Cyclophosphamide and Granulocyte-Colony Stimulating Factor (G-CSF) were administrated during mobilization in 8 patients and G-CSF alone in 1 patient. Data was missing in 1 patient. Conditioning regimen was Melphalan 200mg/m² in 5 patients, BEAM (BCNU-Etoposide-Aracytine-Melphalan) in 3 patients, Cyclophosphamide 200 mg/kg plus Anti-Thymocyte-Globulin (ATG) in 2 patients. Median follow-up was 48 months (range 6-252 months). Detailed data are presented in **Table 1**.

### Main Outcome: Response to Treatment

This procedure induced complete remission (CR) in 80%, partial remission in 10% and lack of response in 10% of the patients. Relapse rate was 30% (2 relapses in patients in CR and 1 relapse in the patient in PR) with panuveitis (n=1), aphthosis (n=2) and arthralgia (n=1) Only one patient did not respond with aHSCT at 6 months and was treated with allogeneic hematopoietic stem cell transplantation (HSCT). At the most recent follow-up, 60% of the patients were in CR without any treatment (including corticosteroids), three patients relapsed at 24, 37 and 48 months (**Table 2**).

### Secondary Outcomes

During aHSCT (before day 100), two patients had a fever of unknown origin, two patients had infectious complications (pneumonia) and in addition to pneumonia a single patient had paroxysmal atrial fibrillation, line-associated deep venous thrombosis and depression. No complications were reported after Day 100. The patient who did not respond to aHSCT was treated with allogeneic HSCT. He died three years after allogeneic transplantation of chronic Graft versus Host Disease complicated by cytomegalovirus infection. No other late death was reported. At one year, 8 patients were in CR and one patient in DDPR. The patient with DDPR had a mild active uveitis. All other involvements improved (skin, eye, gastro-intestinal, cardiac, lung, kidney) but sometimes with irreversible organ damage (decreased visual acuity in patients 1,6,9 and moderate chronic kidney disease in patient 2). In case of neurological involvement (3 patients), 2 patients improved but with residual disability and one patient remained stable. The relapse rate was 3 out 9 (≈ 33%) with only one major relapse. The major involvement was an ocular panuveitis (patient 7) that occurred 48 months after aHSCT leading immediately to introduction of a new treatment. At last follow-up visit, the patient was in DDCR with steroids. Two minor relapses with aphthosis (n=2) and arthralgia (n=1) were reported at 24 and 37 months. Patients experiencing relapse were treated immediately with steroids and azathioprine (AZA) or AZA alone. At last follow-up visit, the patients who relapsed were only treated with AZA and were in DDCR. One of these patients was resistant to AZA before the aHSCT and the patient who relapsed with panuveitis was refractory to steroids before the aHSCT (**Table 2**).

## Discussion

Our study is the first retrospective cohort study conducted in patients with BD who underwent aHSCT. The results showed a high rate of efficacy in heavily pretreated patients or in patients with life-threatening complications with acceptable toxicity profile and no aHSCT-related mortality. Two patients were in CR (*ie* without any treatment) up to 21 years after aHSCT, raising the question of the efficacy of aHSCT as an effective cure for patients with BD refractory to multiple conventional treatments. However, one patient did not respond, one patient had a major relapse and two patients had a minor relapse reflecting some limitations of the procedure. Relapses consisted of the same clinical presentation observed at the onset of the disease. But there was no life-threatening involvement and all relapses were controlled by conventional treatments, whereas patients were refractory to those treatments before aHSCT. This is consistent with observations following aHSCT in refractory Crohn’s Disease and use of anti-TNFα therapy after aHSCT (24) and in rheumatoid arthritis and use of disease-modifying agents after aHSCT (25), where there is renewed sensitivity to drugs associated with the “immune reset” after aHSCT.

Such “immune reset” after aHSCT may explain the efficacy in BD. Although no data are available in BD, this concept is well described in other auto-immune diseases treated with aHSCT, and the mechanisms seem to be similar whatever the disease (26). The destruction of the aberrant immune system leads to restoration of a new naïve and self-tolerant immune system (27–30). Renewal of T-cell repertoire is well documented in rheumatic diseases like systemic sclerosis or systemic lupus erythematosus with the disappearance of T-cell autoimmune clones (31) and of the pre-existing serum auto-antibodies (32). A functional renewal of regulatory T cells (33) and diversification of the T-cell receptor (TCR) repertoire (30) are also associated with favorable outcomes in patients with severe resistant autoimmune and inflammatory diseases treated with aHSCT (34).

Significantly, our results are congruent with published cases showing a high rate of efficacy (16, 19–22). By selecting the patients from EBMT registry with the inclusion of consecutive patients, this study avoided publication bias, i.e. reporting only success and not failure. Furthermore, this study is the first focusing on aHSCT in BD. Other studies had no more than 2 cases as they were focused on primary vasculitis, including BD among others (16, 22), with shorter follow-up (16, 19–22, 35).

tThis study has limitations including retrospective data collection (but missing data were rare, and they pertained to secondary data) and possible under-reporting of aHSCT for BD in the EBMT registry (the 2 cases extracted from the literature illustrate this issue (20), despite the obligation of full EBMT member centers to report all cases). This bias is likely to be independent from aHSCT outcome. The literature search improved sensitivity but may have been affected by publication bias. Another bias was the heterogeneity of patients characteristics at baseline (especially in terms of organ involvements), reflecting the absence of consensus or guidelines on the indication of aHSCT in BD. The long term risk/benefit balance of aHSCT in BD should be evaluated in prospective studies, ideally randomized controlled trials of anti-TNFα therapy refractory patients against other standard of care. But it would be unlikely to be feasible due to the rare incidence of patients with this severity of BD. More pragmatically, ongoing registry-based analyses may help to define the role of aHSCT in BD and further inform transplant technique and patient selection to optimize patient outcomes.

In conclusion, based on our data, aHSCT has an acceptable safety profile and represents a feasible and relatively effective procedure in severe and conventional treatment-resistant cases of BD. It should be considered as a treatment option for patients refractory to anti-TNFα with high disease burden. Further data ideally from randomized controlled trials are warranted.

## Data Availability Statement

The raw data supporting the conclusions of this article will be made available by the authors, without undue reservation.

## Ethics Statement

Ethical review and approval was not required for the study on human participants in accordance with the local legislation and institutional requirements. The patients/participants provided their written informed consent to participate in this study.

## Author Contributions

Substantial contributions to the conception or design of the work; or the acquisition, analysis, or interpretation of data for the work: ALL. Drafting the work or revising it critically for important intellectual content: ALL. Agreement to be accountable for all aspects of the work in ensuring that questions related to the accuracy or integrity of any part of the work are appropriately investigated and resolved: ALL. All authors contributed to the article and approved the submitted version.

## Funding

No specific funding was received from any bodies in the public, commercial or not-for-profit sectors to carry out the work described in this article.

## Conflict of Interest

JS declares previous honoraria for speaking at educational events from Sanofi, Jazz, Janssen, Actelion, Mallinckrodt and Gilead, and is a member of a trial IDMC for Kiadis Pharma (none directly related to the subject of this analysis).

The remaining authors declare that the research was conducted in the absence of any commercial or financial relationships that could be construed as a potential conflict of interest.
